# Microcirculatory perfusion disturbances in septic shock: results from the ProCESS trial

**DOI:** 10.1186/s13054-018-2240-5

**Published:** 2018-11-20

**Authors:** Michael J. Massey, Peter C. Hou, Michael Filbin, Henry Wang, Long Ngo, David T. Huang, William C. Aird, Victor Novack, Stephen Trzeciak, Donald M. Yealy, John A. Kellum, Derek C. Angus, Nathan I. Shapiro, for the ProCESS investigators, Derek C. Angus, Amber E. Barnato, Tammy L. Eaton, Elizabeth Gimbel, David T. Huang, Christopher Keener, John A. Kellum, Kyle Landis, Francis Pike, Diana K. Stapleton, Lisa A. Weissfeld, Michael Willochell, Kourtney A. Wofford, Donald M. Yealy, Erik Kulstad, Hannah Watts, Arvind Venkat, Peter C. Hou, Anthony Massaro, Siddharth Parmar, Alexander T. Limkakeng, Kori Brewer, Theodore R. Delbridge, Allison Mainhart, Lakhmir S. Chawla, James R. Miner, Todd L. Allen, Colin K. Grissom, Stuart Swadron, Steven A. Conrad, Richard Carlson, Frank LoVecchio, Ednan K. Bajwa, Michael R. Filbin, Blair A. Parry, Timothy J. Ellender, Andrew E. Sama, Jonathan Fine, Soheil Nafeei, Thomas Terndrup, Margaret Wojnar, Ronald G. Pearl, Scott T. Wilber, Richard Sinert, David J. Orban, Jason W. Wilson, Jacob W. Ufberg, Timothy Albertson, Edward A. Panacek, Sohan Parekh, Scott R. Gunn, Jon S. Rittenberger, Richard J. Wadas, Andrew R. Edwards, Matthew Kelly, Henry E. Wang, Talmage M. Holmes, Michael T. McCurdy, Craig Weinert, Estelle S. Harris, Wesley H. Self, Diane Dubinski, Carolyn A. Phillips, Ronald M. Migues

**Affiliations:** 1 Department of Emergency Medicine and Center for Vascular Biology Research, Beth Israel Deaconess Medical Center, 1 Deaconess Road, CC2-W, Boston, MA 02215 USA; 2 Department of Emergency Medicine, Brigham and Women’s Hospital, Boston, MA USA; 3 Department of Emergency Medicine, Massachusetts General Hospital, Boston, MA USA; 4 Department of Emergency Medicine, University of Alabama at Birmingham, Birmingham, AL USA; 5 Division of General Medicine, Department of Medicine, Beth Isarel Deaconess Medical Center, Boston, MA USA; 6 Department of Critical Care Medicine, University of Pittsburgh School of Medicine, Pittsburgh, PA USA; 7 Division of Molecular Medicine, Department of Medicine, and Center for Vascular Biology Research, Beth Israel Deaconess Medical Center, Boston, MA USA; 8 Clinical Research Center, Soroka University Medical Center, Be’er-Sheva, Israel; 9 Center for Critical Care Services, Cooper University Hospital, Camden, NJ USA; 10 Department of Emergency Medicine, University of Pittsburgh School of Medicine, Pittsburgh, PA USA

**Keywords:** Sepsis, Microcirculation, Pathophysiology, Mortality

## Abstract

**Background:**

We sought to determine the effects of alternative resuscitation strategies on microcirculatory perfusion and examine any association between microcirculatory perfusion and mortality in sepsis.

**Methods:**

This was a prospective, formally designed substudy of participants in the Protocolized Care in Early Septic Shock (ProCESS) trial. We recruited from six sites with the equipment and training to perform these study procedures. All subjects were adults with septic shock, and each was assigned to alternative resuscitation strategies. The two main analyses assessed (1) the impact of resuscitation strategies on microcirculatory perfusion parameters and (2) the association of microcirculatory perfusion with 60-day in-hospital mortality. We measured sublingual microcirculatory perfusion using sidestream dark field in vivo video microscopy at the completion of the 6-h ProCESS resuscitation protocol and then again at 24 and 72 h.

**Results:**

We enrolled 207 subjects (demographics were similar to the overall ProCESS cohort) and observed 40 (19.3%) deaths. There were no differences in average perfusion characteristics between treatment arms. Analyzing the relationship between microcirculatory perfusion and mortality, we found an association between vascular density parameters and mortality. Total vascular density (beta = 0.006, *p* < 0.003), perfused vascular density (beta = 0.005, *p* < 0.04), and De Backer score (beta = 0.009, *p* < 0.01) were higher overall in survivors in a generalized estimating equation model, and this association was significant at the 72-h time point (*p* < 0.05 for each parameter).

**Conclusions:**

Microcirculatory perfusion did not differ between three early septic shock treatment arms. We found an association between microcirculatory perfusion parameters of vascular density at 72 h and mortality.

**Trial registration:**

ClinicalTrials.gov, NCT00510835. Registered on August 2, 2007.

**Electronic supplementary material:**

The online version of this article (10.1186/s13054-018-2240-5) contains supplementary material, which is available to authorized users.

## Background

Patients with sepsis have high morbidity, mortality, and care costs. Improving outcomes requires an enhanced understanding of the complex pathophysiology of the disease. Organ dysfunction and multisystem organ failure are common precursors to death in sepsis. The smallest blood vessels of the microcirculation (< 20 μm in diameter) are the principal sites of gas and nutrient exchange between blood and underlying tissues [1]. Microcirculatory perfusion disturbances represent a direct physiologic link to multisystem organ dysfunction. Microcirculatory perfusion disturbances represent a potential universal link across organs and could alter insights and care.

Possible causes of microcirculatory perfusion alterations in sepsis include endothelial cell dysfunction, glycocalyx degradation, increased leukocyte adhesion, microthrombus formation, rheological abnormalities, altered local perfusion pressures due to regional redistribution of blood flow, and functional shunting. The microcirculation is impaired in sepsis [2–14], and the defect in perfusion may be therapeutically reversed [4, 15–17]. With the advent of handheld in vivo imaging modalities, it is possible to visualize the sublingual microcirculation in human patients at the bedside [1, 18–20].

Microcirculatory perfusion represents the combination of the density of vessels available to provide nutrients (e.g., oxygen) carrying blood to the organs and the flow rates of that blood. Clinical studies suggest that persistent microcirculatory alterations refractory to resuscitation are prognostic of fatal outcome [3, 4, 8–10, 12–15, 17, 21, 22] independent of systemic variables and oxygen-derived variables [4, 8, 17]. Changes in microvascular perfusion may occur in the *absence* of global hemodynamic perturbations (i.e., low blood pressure/cardiac output), indicating that these alterations are intrinsic to the microcirculation.

Prior studies of microcirculatory perfusion disturbances in sepsis and septic shock have typically been either limited in size [3, 8, 10, 14, 17, 23] or initiated in the intensive care unit (ICU) well after the onset of sepsis. For this project, we studied patients enrolled in the Protocolized Care for Early Septic Shock (ProCESS) study, a randomized clinical trial of three alternative resuscitation strategies that included the administration of fluids, vasopressors, blood, and dobutamine (each with a previously published impact on the microcirculation). We sought to (1) determine the effects of alternative resuscitation strategies on microcirculatory perfusion disturbances in early septic shock and (2) study the association between microcirculatory perfusion disturbances over the first 72 h of resuscitation and 60-day in-hospital mortality in early septic shock.

## Methods

### Study aim, design, and setting design

We enrolled a subpopulation of subjects participating in the ProCESS trial, a patient-level randomized multicenter interventional trial of alternative resuscitation strategies in emergency department (ED) early septic shock [24]. In the ProCESS trial, subjects with sepsis and hypoperfusion (*see* enrollment criteria below) randomly received one of three resuscitation strategies: early goal-directed therapy (EGDT) as described by Rivers et al. [25] and delivered by a study team, a strategy of noninvasive protocolized care delivered by a study team, or usual care absent any protocol or prompts and delivered by the clinical team [24]. Six hospital sites participated in this ancillary study to the ProCESS trial.

The primary outcome was in-hospital mortality by day 60. We registered the current trial and ProCESS with ClinicalTrials.gov under the identifiers NCT00793442 and NCT00510835, respectively, and the Beth Israel Deaconess Medical Center Committee for Clinical Investigations and each site’s institutional review board approved the design. Each subject or legal representative gave written informed consent.

### Participants

ProCESS trial subjects [24] all had (1) suspected infection in the ED; (2) at least two systemic inflammatory response syndrome criteria [26]; and (3) refractory hypotension, defined as a systolic blood pressure < 90 mmHg despite an intravenous fluid challenge of at least 1 L of crystalloids or evidence of tissue hypoperfusion (blood lactate concentration ≥ 4 mmol/L). They were enrolled as a convenience sample at sites participating in this ancillary study. An additional exclusion criterion for this specific ancillary study was the inability to tolerate study procedures due to an oxygen requirement; for example, a patient on a nonrebreather mask could not tolerate removing the mask to perform the microcirculation image collection.

### Demographics and clinical data collection

We collected information on patient demographics, comorbid illnesses, etiologies of infection, and treatments. We also collected macrocirculatory perfusion parameters.

### Microcirculatory video microscopy image capture and management

We visualized and recorded the sublingual microcirculation using sidestream dark field video microscopic imaging (MicroScan; MicroVision Medical, Inc., Amsterdam, The Netherlands) as previously described [18, 20, 27]. We captured videos at 6, 24, and 72 h after enrollment. We specifically did not attempt microcirculation imaging during the initial 6-h resuscitation period so as not to affect the main trial’s intervention. We uploaded stored video clips to the central laboratory using both a proprietary file transfer software (Studymaker File Manager; Studymaker, Newton, MA, USA) and a cloud storage service (Dropbox; Dropbox, Inc., San Francisco, CA, USA) [18, 27, 28].

### Site training

Study team participants received instruction on the operation and use of the MicroScan device primarily through live training during individualized training sessions provided at their institution. Instruction consisted of (1) overview of the pathophysiology of microcirculatory derangements in sepsis; (2) introduction to the MicroScan device as well as its setup and operation; and (3) a detailed presentation of the technique for image acquisition, including positioning, lighting, focus, and image recording. All participants then practiced using healthy volunteers. We focused on the five points for image acquisition outlined by the microcirculation consensus conference as key for quality image analysis: (1) obtain five sample sites per subject, (2) avoid pressure artifacts, (3) eliminate secretions, (4) adequate focus and brightness adjustment, and (5) recording quality [29]. Study team members were required to demonstrate proficiency in obtaining images by adequately recording a video to “pass” training.

### Image analysis

We processed MicroScan video files to enhance contrast, edited to visually stable sequences of 3–10 s in duration, and evaluated for image quality using the method described by Massey et al. [27]. We sorted video clips using an ordinal quality score with up to three clips selected at each of the three time points, and then we assigned a random identifier before further microvascular analysis using AVA 3.1 software (MicroVision Medical BV). We did not rely on automated vessel identification; instead, vessel centerlines and lumen boundaries were drawn using manual tools in AVA. Vessel classification was done using sizing, with small, medium, and large vessels having lumen diameters < 20 μm, 20–50 μm, and > 50 μm, respectively. All of the microcirculatory parameters reported were from analyses of small vessels (< 20 μm) because capillaries are the principal sites of oxygen exchange and fall into the small vessel size range.

### Scoring the images

For microcirculatory perfusion image analysis, we followed the methods of the 2007 consensus conference on evaluation of the microcirculation [29]. We report the suggested measures of proportion of perfused vessels (PPV), microcirculatory flow index (MFI), De Backer score, total vascular density (TVD), perfused vascular density (PVD), and heterogeneity index (Table 1) [29]. PPV is the perfused linear density of small vessels in a field of view, computed as the length of perfused small vessels divided by the total length of small vessels visualized [18, 30]. MFI calculations followed the semiquantitative technique described by Spronk et al. [15] (0 = absent flow; 1 = sludging/noncontinuous flow; 2 = moderate flow; 3 = normal/brisk flow), determining the mode for each quadrant and averaging over the four quadrants to yield the MFI. De Backer score calculations used the line crossing technique [31]. We calculated TVD by quantifying the total density of small vessels within the field of view and calculated PVD by measuring the density of perfused small vessels within the field of view. Heterogeneity index calculations used the highest MFI over the four quadrants, subtracting the lowest MFI over the four quadrants and dividing by the mean MFI [8, 29]. For the perfusion parameters, we considered any vessel segment with a flow score greater than or equal to 2 (moderate/normal flow) as perfused. After vessel detection, we used the AVA software to make the calculations described above and quantify vessel length. We calculated the PPV by dividing the total vessel length by the perfused vessel length.

**Table 1:** Summary of microcirculatory parameters quantitative metrics

Name	Abbreviation	Description
Microcirculatory flow index	MFI	A qualitative assessment of flow over quadrants. Predominant (mode) flow velocity of visualized vessels is determined for each image quadrant. MFI is computed as the average of the predominant flow classification of small vessels over the four image quadrants.
Heterogeneity index	HETERO	A measure of flow heterogeneity. Computed as the maximum MFI quadrant value minus minimum MFI quadrant value divided by mean MFI.
Total vascular density	TVD	A measure of all vessels over the field of view. Computed as total length of small vessels divided by total area of field of view.
Proportion of perfused vessels	PPV	A measure of the lineal proportion of perfused vessels. Computed as total length of perfused small vessels (semiqualitative velocity score 2 or greater)^a^ divided by total length of small vessels.
Perfused vascular density	PVD	A measure of the perfused vessel lineal density. Computed as proportion of perfused vessels multiplied by total vessel density.
De Backer score	De Backer	An alternate measure of total vessel density. A grid is formed using three equally spaced vertical and three horizontal lines over image area. Computed as number of vessels crossing grid lines divided by the total length of the lines.

^a^ Semiquantitative velocity scoring: 0 = absent flow; 1 = noncontinuous/sluggish flow; 2 = moderate flow; 3 = normal continuous/brisk flow [15, 29]

A summary of microcirculatory flow metrics following the recommendations of a consensus report [29]

### Statistical analysis

We assessed for normality and proceeded with parametric or nonparametric testing, with two-tailed alpha set at 0.05. The microcirculatory perfusion parameters of interest were PPV, MFI, De Backer score, TVD, PVD, and heterogeneity index. For any effect of the randomly assigned treatment strategies on the different microcirculatory perfusion parameters, we report the mean (plus SD) or median [IQR] for each of the microcirculatory perfusion parameters by study arm. For differences in microcirculatory perfusion parameters by mortality endpoint, we report the differences in demographics, comorbidities, and sepsis etiologies between survivors and nonsurvivors.

For the analysis of an association between assigned treatment arm and microcirculation parameters, we used a generalized estimating equation (GEE) with a subject being a cluster (nonstructured correlation matrix) with pairwise comparisons with adjustment for multiple comparisons (LSD). For the normally distributed parameters of TVD, PVD, and De Backer score, we used linear models within GEE; for MFI and PPV, which were skewed, we applied a gamma distribution with log-link functions. For the heterogeneity index parameter, we chose a Tweedie distribution. Within each model, we assessed the interaction between the microcirculation parameter and time point. Because the interaction between the microcirculation parameters and time was not significant in all models, we report the results based on the models without the interactions included. For the analysis of the association between microcirculation parameters and 60-day in-hospital mortality, we used GEE with subject being a cluster (nonstructured correlation matrix) using logistic regression with robust estimator and unstructured correlation matrix adjusted for Charlson comorbidity index and age.

Finally, we compared the mean value of macrocirculatory parameters among survivors and nonsurvivors. We used GEE models to evaluate the association between the macrocirculatory and microcirculatory parameters adjusted for age and Charlson comorbidity index score.

### Sample size

To estimate sample size for the impact of the resuscitation protocols on microcirculatory perfusion based on pilot data, we used an estimated mean of MFI (considered the “main” microcirculatory perfusion parameter reported at the time) at 2.3 ± 0.6 for standard care, expecting subjects with protocolized goal-directed resuscitation to have a mean MFI 20% greater than this mean. At a power of 0.9 to detect a mean difference of 0.46, the estimated sample size was 114. For the association between MFI and mortality, we estimated that the OR of mortality would increase by 50% per 0.5 of an SD decrease in the MFI (thus an OR of 1.5 for a 0.5-SD decrease). For type I error of 0.05 and a power of 0.9, the estimated sample size was 115 subjects. Because the current reported trial was ongoing and part of a larger parent trial collecting biomarkers for up to 600 patients, in consultation with the data and safety monitoring board, we continued enrollment to surpass our sample size estimates to increase our overall power.

## Results

From the parent trial of 1341 patients, we enrolled 225 (16.8%) into this substudy, and 207 (92%) subjects had adequate images and were analyzed. A total of 1244 images from 439 time points were included (an average of 2.8 videos per patient time point).

### Patient demographics

Our study subjects had a mean age of 61 years (SD 16), 73% were white, and 20% were African American (Table 2). Similar to prior studies, the prevalence of comorbid illness was high, including high rates of hypertension, diabetes, and cancer. Pneumonia was the most common underlying etiology of sepsis. The baseline Sequential Organ Failure Assessment score was 7.7 (SD 3.8). Among the 207 participants, there were 40 deaths, for an overall mortality rate of 19.3%. The population enrolled in this ancillary study was very similar to and representative of the population enrolled in the ProCESS study overall (Additional file 1: Table S1).

**Table 2:** Demographics

Characteristic	Entire cohort (*N* = 207)	Lived (*n* = 167)	Died (*n* = 40)
Age, years, mean (SD)	60.9 (15.6)	59.44 (15.95)	67.23 (12.37)
Female sex	106 (51)	87 (52)	22 (55)
Race			
White	152 (73)	124 (74)	28 (70)
Black or African American	41 (20)	31 (19)	10 (25)
Asian	8 (3.9)	7 (4.2)	1 (2.5)
Other	5 (2.4)	4 (2.4)	1 (2.5)
Ethnicity			
Non-Hispanic	189 (91)	149 (89)	40 (100)
Hispanic	18 (8.7)	18 (11)	0 (0.00)
Domicile prior to admission			
Non-nursing home	185 (89)	149 (89)	36 (90)
Nursing home	22 (11)	18 (11)	4 (10)
Chronic conditions			
Charlson comorbidity index score, mean (SD)	3.1 (2.7)	2.77 (2.5)	4.35 (3.3)
Hypertension	123 (59)	95 (57)	28 (70)
Diabetes mellitus	64 (31)	48 (29)	16 (40)
Chronic respiratory disease	54 (26)	43 (26)	11 (28)
Cancer	52 (25)	38 (23)	14 (35)
Dialysis-dependent renal impairment	14 (6.8)	12 (7.1)	2 (5.0)
Congestive heart failure	29 (14)	22 (13)	7 (18)
Prior myocardial infarction	22 (11)	15 (9.0)	7 (18)
Cerebral vascular disease	21 (10)	17 (10)	4 (10)
Peripheral vascular disease	21 (10)	14 (8.4)	7 (18)
Chronic dementia	14 (6.8)	10 (6.0)	4 (10)
Hepatic cirrhosis	17 (8.2)	11 (6.6)	6 (15)
Peptic ulcer disease	11 (5.3)	9 (5.4)	2 (5.0)
AIDS and related syndromes	1 (1.9)	2 (1.2)	2 (5.0)
Source of sepsis			
Pneumonia	69 (33.3)	57 (34.)	12 (30)
Urosepsis	40 (19.3)	32 (19)	8 (20)
Infected, source unknown	19 (9.2)	16 (9.6)	3 (7.5)
Intra-abdominal infection	33 (16)	22 (13)	11 (28)
Skin and soft tissue infections	14 (6.8)	13 (7.8)	1 (2.5)
Catheter-related infection	14 (6.8)	11 (6.6)	3 (7.5)
Central nervous system	2 (1.0)	2 (1.2)	0 (0.0)
Endocarditis	4 (1.9)	4 (2.4)	0 (0.0)
Other	9 (4.4)	8 (4.8)	1 (2.5)
Considered after review not to be infected	3 (1.5)	2 (1.2)	1 (2.5)
Baseline SOFA score, mean (SD)	7.7 (3.8)	6.96 (3.4)	10.83 (3.8)

*AIDS* Acquired immunodeficiency syndrome, *SOFA* Sequential Organ Failure Assessment

Data are number (%) unless otherwise noted

### Microcirculatory perfusion image analysis

There were 225 total patients with images obtained across the 6-, 24-, and 72-h time points, with attempts made during 552 (86%) of the available time points (Additional file 2: Table S2). Among the images processed and analyzed, 439 (80%) of the time points had images of suitable quality for analysis. The success of image acquisition by time point was as follows: 6 h (170 of 205; 83%), 24 h (149 of 200; 75%), and 72 h (120 of 147; 82%). Pressure artifacts were the most common reason for not passing our quality check (40% of failures), followed by content artifacts (30% of failures).

### Effect of resuscitation strategy on microcirculatory perfusion

There were no differences in clinical outcome between resuscitation strategies in the parent ProCESS trial [24]. Similarly, there were no differences in the microcirculatory perfusion parameters of PPV, De Backer score, TVD, PVD, or heterogeneity index for each time point between the three treatment arms (Table 3 and Additional file 3: Table S3). MFI did show a difference between the three arms with impaired perfusion in the EGDT group; however, the mean difference was quite small, which limits clinical impact. Because there were no meaningful differences in microcirculatory perfusion patterns between the groups, the treatment groups were pooled for subsequent analyses.

**Table 3:** Analysis of an association between arms and microcirculation parameters

	EGDT vs. control		Noninvasive vs. control	
	Beta	*p* Value	Beta	*p* Value
Total vascular density (mm/mm^2^)	− 0.92	0.16	0.23	0.72
Perfused vascular density (mm/mm^2^)	− 1.29	0.07	− 0.24	0.74
De Backer score	− 0.42	0.30	0.05	0.91
Heterogeneity index	0.11	0.09	0.02	0.71
Microcirculatory flow index	**− 0.06**	**< 0.02**	− 0.02	0.36
Proportion perfused vessels (%)	− 0.05	0.09	0.00	0.94

*EGDT* Early goal-directed therapy

In this analysis, a generalized estimating equation model adjusted for age and Charlson comorbidity index score was used to assess differences in microcirculatory flow parameters between treatment arms. The only parameter found to be statistically significant was microcirculatory flow index, which was lower in the early goal-directed therapy group compared with control; however, the small difference limits the clinical significance of this finding. There was no significant difference across all other parameters when comparing each of the arms

Boldface indicates *p*<0.05

### Association between microcirculatory perfusion parameters and mortality

**Fig. 1:**
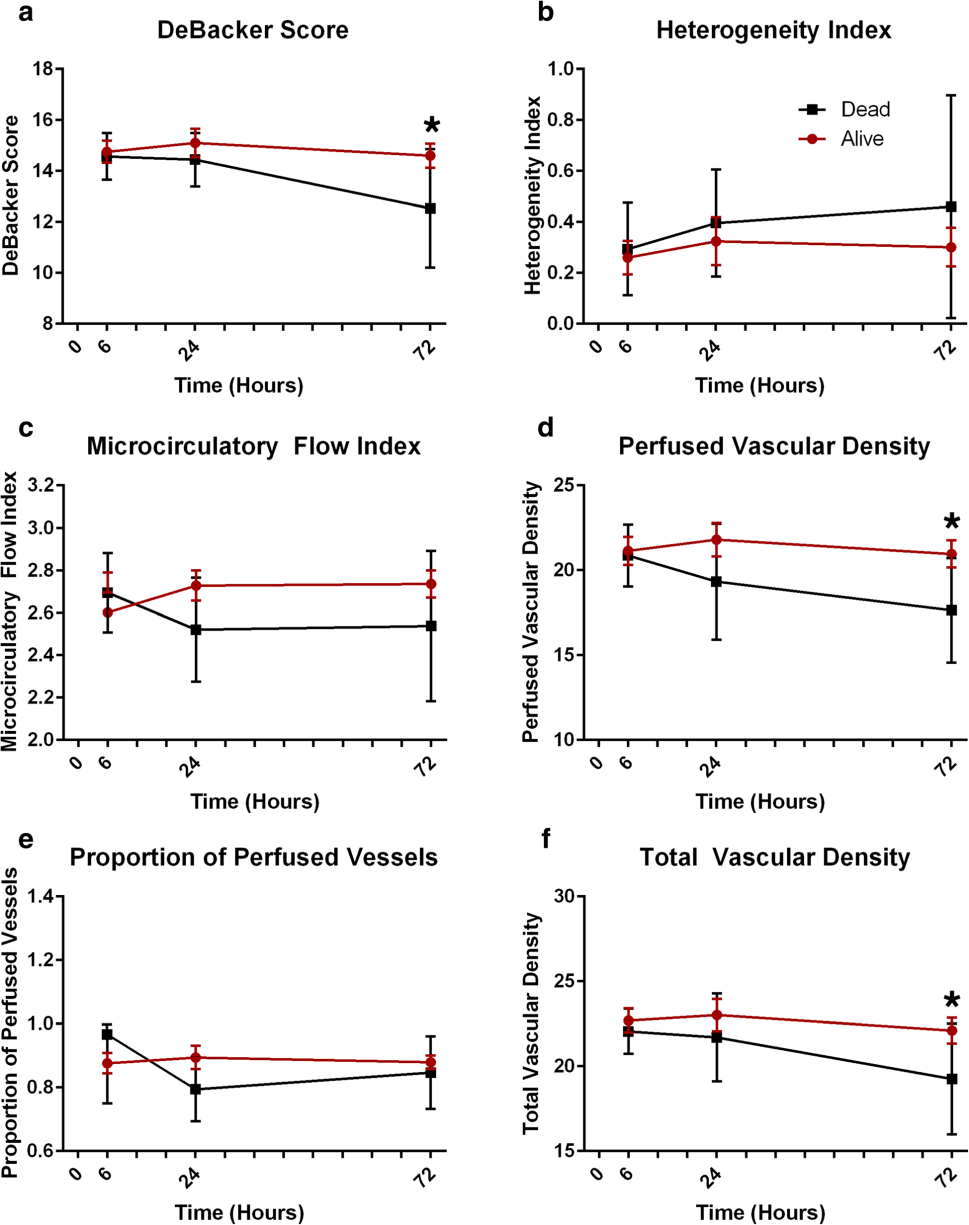
**a**–**f** Changes in the microcirculatory perfusion parameters over time. These parameters include De Backer score (**a**), heterogeneity score (**b**), microcirculatory flow index (**c**), perfused vascular density (**d**), proportion of perfused vessels (**e**), and total vascular density (**f**). *Significance at a level of *p* < 0.05

The microcirculation parameters of TVD (beta = 0.006, *p* < 0.003), PVD (beta = 0.005, *p* < 0.04), and De Backer (beta = 0.009, *p* < 0.01) scores were higher in survivors in a GEE model that considered each parameter at all time points (Table 4 and Fig. 1). This indicates an impairment of the microcirculation in those who eventually died. These differences were driven primarily by the 72-h TVD, PVD, and De Backer scores, which were lower among nonsurvivors at 72 h. The microcirculation parameters of MFI, PPV, and heterogeneity index were similar among survivors and nonsurvivors at each time point and in the overall models (Table 4 and Fig. 1).

**Table 4:** Differences in microcirculation parameters between survivors and nonsurvivors

Microcirculation parameter	Alive at discharge			Dead at discharge			Overall model Alive vs. dead	
	6 h *n* = 138	24 h *n* = 128	72 h *n* = 108	6 h *n* = 32	24 h *n* = 21	72 h n = 12	Beta	*p* Value*
TVD, mean ± SD	22.6 ± 4.2	23.0 ± 5.4	**22.1 ± 4.0**	21.1 ± 3.7	21.7 ± 5.7	**19.3 ± 5.1**	**0.006**	**< 0.003**
PVD, mean ± SD	21.1 ± 4.8	21.8 ± 5.7	**21.0 ± 4.2**	20.8 ± 4.9	19.3 ± 7.5	**17.6 ± 4.8**	**0.005**	**< 0.04**
De Backer score, mean ± SD	14.8 ± 2.6	15.1 ± 3.2	**14.6 ± 2.5**	14.5 ± 2.6	14.4 ± 2.3	**12.5 ± 3.7**	**0.009**	**< 0.01**
Heterogeneity index, median [IQR]	0.08 [0.00–0.43]	0.18 [0.00–0.46]	0.17 [0.00–0.49]	0.00 [0.00–0.51]	0.27 [0.00–0.67]	0.29 [0.00–0.60]	− 0.02	0.54
MFI, median [IQR]	2.92 [2.59–3.00]	2.85 [2.61–3.00]	2.88 [2.51–3.00]	2.96 [2.56–3.00]	2.67 [2.18–3.00]	2.71 [2.25–3.00]	0.05	0.33
PPV, median [IQR]	0.90 [0.83–0.97]	0.90 [0.85–0.96]	0.91 [0.83–0.95]	0.91 [0.82–0.95]	0.91 [0.83–0.95]	0.92 [0.69–1.00]	− 1.16	0.46

*Abbreviations: MFI* Microcirculatory flow index, *PPV* Proportion of perfused vessels, *PVD* Perfused vascular density, *TVD* Total vascular density

*In **bold**, *p* < 0.05 for pairwise comparisons at a given time point

The mean (± SD) and median [IQR] values are shown for each microcirculation parameter at 6-, 24-, and 72-h time points for survivors and nonsurvivors. TVD, PVD, and De Backer scores are lower in nonsurvivors, indicating an impaired microcirculation. In generalized estimating equation models, TVD, PVD, and De Backer scores were lower overall in nonsurvivors than in survivors

### Association between macrocirculatory and microcirculatory parameters

In an exploratory analysis, we examined the relationship between macrocirculatory parameters and microcirculatory parameters. First, we assessed differences in macrocirculatory and microcirculatory parameters among survivors and nonsurvivors in aggregate (Table 5) and found survivors to have a higher driving pressure (MAP − CVP) and lower serum lactate, whereas there were no differences in the other parameters. In an adjusted analysis, only increased CVP was associated with higher TVD, De Backer score, and heterogeneity index, whereas there was an unexpected correlation between CVP and lower MFI score as well as between mean arterial pressure and lower TVD (Table 6). We identified a weak correlation between CVP and some flow (heterogeneity index) and density parameters (TVD and De Backer score); however, overall, the data do not show a meaningful association between macrocirculatory and microcirculatory parameters.

**Table 5:** Average macrocirculatory parameters among survivors and nonsurvivors

	Dead *n* = 40	Alive *n* = 167	*p* Value
Systolic blood pressure (mmHg)	104.9 ± 14.3	108.7 ± 14.9	0.20
Diastolic blood pressure (mmHg)	56.8 ± 9.5	59.3 ± 8.4	0.14
Mean arterial pressure (mmHg)	72.8 ± 9.5	75.8 ± 9.3	0.11
Heart rate (beats per minute)	98.2 ± 19.1	94.3 ± 17.7	0.27
Central venous pressure (mmHg)	14.0 ± 5.3	11.8 ± 5.2	0.11
Scvo_2_	76.9 ± 6.1	73.4 ± 10.7	0.32
Driving pressure (mmHg)	56.1 ± 5.4	63.2 ± 9.7	0.004
Lactate (mmol/dl)	4.7 ± 4.5	1.7 ± 1.2	< 0.001

*Scvo*_*2*_ Central venous oxygen saturation

Table shows the average macrocirculatory flow parameters based on survival status incorporating multiple time points

**Table 6:** Relationship between macrocirculatory and microcirculatory parameters

	MAP	SBP	DBP	Heart rate	CVP	MAP − CVP	Lactate
TVD (mm/mm^2^)	**− 0.038 (0.018)**	− 0.076 (0.34)	0.075 (0.30)	− 0.01 (0.396)	**0.32 (0.031)**	− 0.15 (0.17)	− 0.071 (0.59)
PVD small (mm/mm^2^)	0.022 (0.61)	0.032 (0.26)	−0.004 (0.93)	− 0.005 (0.738)	− 0.011 (0.91)	0.024 (0.56)	− 0.457 (0.07)
De Backer score	− 0.034 (0.75)	− 0.032 (0.58)	− 0.023 (0.83)	− 0.002 (767)	**0.23 (0.048)**	− 0.065 (0.47)	− 0.188 (0.27)
Heterogeneity index	− 0.001 (0.92)	− 0.004 (0.59)	**0.16 (< 0.001)**	**− 0.012 (0.02)**	**0.051 (0.015)**	− 0.01 (0.40)	0.004 (0.91)
MFI	0.00 (0.89)	0.00 (0.68)	0.00 (0.91)	0.001 (0.738)	**− 0.028 (< 0.001)**	0.003 (0.075)	− 0.013 (0.057)
PPV (%)	0.001 (0.75)	0.00 (0.75)	0.00 (0.79)	0.001 (0.301)	− 0.005 (0.11)	0.001 (0.39)	− 0.022 (0.24)

*Abbreviations: MAP* Mean arterial pressure, *SBP* Systolic blood pressure, *DBP* Diastolic blood pressure, *CVP* Central venous pressure, *TVD* Total vascular density, *PVD* Perfused vascular density, *MFI* Microcirculatory flow index, *PPV* Proportion of perfused vessels

We used generalized estimating equation models to evaluate the association between the macrocirculatory and microcirculatory parameters adjusted for age and Charlson score. For TVD, PVD, and De Backer scores that were normally distributed, we used linear models as link functions; for MFI, PPV due to the skewness, we applied gamma distribution with log-link functions and for heterogeneity index, Tweedie distribution was chosen due to the abundance of observations at 0. Beta with (*p* value) is shown

Boldface indicates *p*<0.05

## Discussion

Prior studies have shown a relationship between early microcirculatory indices and survival during the resuscitation phase of therapy [3, 4, 8, 22]. De Backer et al. [22] found that decreased microcirculatory perfusion as measured by PPV, PVD, and MFI was associated with mortality. They reported that the PPV parameter was the strongest predictor of mortality and that this association was maintained in multiple logistic regression models for both early (< 24 h) and late (≥ 24 h) time points. The overall AUCs for mortality for PPV and PVD were 0.82 and 0.74, respectively. In a similar study, Trzeciak et al. [8] investigated 26 patients with sepsis in the ED and found that impaired flow and increased heterogeneity of flow were significantly disturbed features of the microcirculation in nonsurvivors compared with survivors. Furthermore, in a study of 49 ICU patients in septic shock, Sakr et al. [4] found that there was no difference in microcirculatory perfusion parameters at the onset of shock, but survivors were able to restore their microcirculatory perfusion as indicated by significant differences in PPV, whereas nonsurvivors had persistently impaired perfusion.

Our findings support a role for microcirculatory perfusion disturbances in sepsis pathophysiology. However, we found a variable association between the different microcirculatory perfusion parameters and mortality. Our study approach and findings support the Sakr et al. [4] study findings, where the initial adequacy of microcirculatory perfusion was not as important as the ability to recover microcirculatory perfusion over time to predict mortality. Our study also demonstrated that parameters of vessel density (TVD and De Backer score) and density of vessels with flow (PVD) were more highly associated with outcome than parameters of flow alone, such as our primary outcome of MFI, or PPV, in which there were not significant differences. Our study occurred during a clinical trial of resuscitation therapy in which patients were treated with a structured resuscitation strategy or with usual care that was aggressive but different from the structured approaches, and the latter performed in a similar fashion for most outcomes. This usual care in the parent trial may differ from previous “wild-type” treatments in observational trials. Although our study supports an association between impaired microcirculatory perfusion and mortality in sepsis, our findings suggest that this association is perhaps less robust than others have suggested.

As highlighted above, there is no clear consensus on which microcirculatory perfusion parameter is most important. For example, De Backer et al. previously found PPV to be the parameter most strongly associated with mortality [3, 22]. In this study, we found the measures of density, namely TVD, PVD, and De Backer score (an estimate of total density), to be associated with mortality when considering all parameters at all time points in a single model and at the 72-h time period. Sakr et al. [4] found PPV to be the most prognostic of outcome [18]. Although the parameters PPV (based on per-vessel quality of flow) and MFI (based on a visual estimate of overall flow quality) have previously been demonstrated to be the most important predictors of outcome, our results support that the density parameters of TVD, PVD, and De Backer score were more tightly associated with mortality and thus perhaps more important. On the other hand, the ability of blood to deliver oxygen is paramount; thus, presence of circulating red blood cells may be more important than the speed at which they are flowing.

There are a number of limitations to this study. First, there is a potential selection bias because we obtained videos only in a subset of subjects; thus, it is possible that those who were more (or less) ill may have had successful image acquisition in a nonrandom fashion. However, our ancillary study cohort with successful image acquisition did have a mortality rate similar to that in the overall trial. Second, there were a number of trained operators across centers but with varying prior experience with microcirculatory flow image acquisition; it is possible that suboptimal image acquisition influenced the results (e.g., pressure artifact; if the operator pushed down too hard, it may have given the false perception of occluded flow). We tried to guard against this through an approach of selecting and including only videos judged as free of such influences. Conversely, our exclusion of a relatively high ratio of videos with presumed pressure artifacts may have contributed to bias. It is possible that pressure artifacts were more likely in patients with more (or less) severe illness. Third, to avoid affecting the ProCESS intervention, we delayed our image acquisition until after the experimental protocol intervention period (the first 6 h after eligibility) was over; it is possible that microcirculation could have differed at baseline. Fourth, other factors, such as chronic diseases or other confounders, may have altered the association of microcirculatory perfusion parameters with mortality. Fifth, we assessed multiple microcirculatory flow and density parameters simultaneously, and it is possible that some significant results were type I errors. Finally, we identified vessels in the images by eye, drew them by hand, and visually estimated the flow rate. It is possible that other automated techniques may yield different results.

Future initiatives should continue to focus on delineating which microcirculatory parameters have the most significant pathophysiologic impact. We found that measures of microvessel density (TVD and De Backer score) and perfusion (PVD) were associated with mortality, whereas measures of flow quality were not. Similarly, software development for analysis, especially at the point of care, may aid future efforts. We used a semiquantitative technique whereby we traced vessels by hand and made empiric estimates on the rate of flow. Reliable automated techniques for vessel identification and flow assessment are still needed.

## Conclusions

We found that the microcirculation in patients in septic shock was not differentially influenced by these three early treatment strategies, which included two protocolized approaches guided by specific physiologic input. There was not an association between microcirculatory perfusion parameters; however, we observed a positive association between microvascular density parameters measured at 72 h and in-hospital mortality by day 60. Conventional resuscitation therapy incompletely normalized microcirculatory perfusion in nonsurvivors. Novel agents that target the restoration of microcirculatory perfusion disturbances may be a promising future therapeutic approach in sepsis.

## Additional files

Additional file 1: **Table S1.** The overall ProCESS population compared with the population enrolled in the microcirculatory flow ancillary study. * *p* < 0.05. (DOCX 17 kb)

Additional file 2: **Table S2.** Microcirculatory perfusion parameters by study arm. This table shows the distribution of patients and mortality rates by study arm included in the ancillary study. Overall, the mortality rates among the groups were similar. (DOCX 13 kb)

Additional file 3: **Table S3.** Microcirculatory perfusion analysis by study arm. In a comparison of study parameters, only MFI was found to have a statistically significant difference between study arms; however, the clinical significance of this small difference is likely minimal. (DOCX 16 kb)

## Abbreviations

AIDS: Acquired immunodeficiency syndrome
CVP: Central venous pressure
DBP: Diastolic blood pressure
ED: Emergency department
EGDT: Early goal-directed therapy
GEE: Generalized estimating equation
ICU: Intensive care unit
MAP: Mean arterial pressure
MFI: Microcirculatory flow index
PPV: Proportion of perfused vessels
ProCESS: Protocolized Care for Early Septic Shock study
PVD: Perfused vascular density
SBP: Systolic blood pressure
Scvo_2_: Central venous oxygen saturation
SOFA: Sequential Organ Failure Assessment
TVD: Total vascular density
