# Supplementary material for: Microcirculatory perfusion disturbances in septic shock: results from the ProCESS trial
Source: Crit Care. 2018 Nov 20;22:308. doi: 10.1186/s13054-018-2240-5 (PMC6245723; doi:10.1186/s13054-018-2240-5)
Supplement: Supplementary file 1 — Table S1. The overall ProCESS population compared with the population enrolled in the microcirculatory flow ancillary study. * p < 0.05. (DOCX 17 kb) [file 13054_2018_2240_MOESM1_ESM.docx]

**Additional file 1: Table S1:** Comparison of Microcirculation Ancillary Cohort Overall ProCESS Cohort

| Characteristic  n (%) unless otherwise noted | ProCESS Cohort  (N= 1341) | Ancillary Cohort  (N=207) |
| --- | --- | --- |
| Age – year,^a^ mean (STD) | 61 (±16.1) | 60.9 (±15.6) |
| Female sex, n (%) | 593 (44) | 106 (51) |
| Race, n (%) |  |  |
| White | 916 (68) | 152 (73) |
| Black or African American | 333 (25) | 41 (20) |
| Asian | 26 (2) | 8 (3.9) |
| Other | 66 (5) | 5 (2.4) |
| Ethnicity n (%)^b^ |  |  |
| Non-Hispanic | 1196 (89) | 189 (91) |
| Hispanic | 143 (11) | 18 (8.7) |
| Domicile prior to admission n (%)^c^ |  |  |
| Non-nursing home | 1128 (84) | 185 (89) |
| Nursing home | 209 (16) | 22 (11) |
| Severity of Illness |  |  |
| APACHE-II, mean (STD) | 20.7 (±7.6) | 20.6 (±7.6) |
| Chronic conditions n (%) unless specified |  |  |
| Charlson comorbidity score, mean (STD) | 2.7 (±2.6) | 3.1 (±2.7) |
| Hypertension | 789 (59) | 123 (59) |
| Diabetes mellitus | 458 (34) | 64 (31) |
| Chronic respiratory disease | 298 (22) | 54 (26) |
| Cancer | 234 (17) | 52 (25) |
| Dialysis dependent Renal impairment | 213 (16) | 14 (6.8) |
| Congestive heart failure | 161 (12) | 29 (14) |
| Prior myocardial infarction | 143 (11) | 22 (10) |
| Cerebral vascular disease | 126 (9) | 21 (10) |
| Peripheral vascular disease | 110 (8) | 21 (10) |
| Chronic dementia | 100 (7) | 14 (6.8) |
| Hepatic cirrhosis | 87 (6) | 17 (8.2) |
| Peptic ulcer disease | 72 (5) | 11 (5.3) |
| AIDS and related syndromes | 38 (3) | 1 (1.9) |
| Source of sepsis |  |  |
| Pneumonia | 443 (33.0) | 69 (33.3) |
| Urosepsis | 284 (21.2) | 40 (19.3) |
| Infected, source unknown | 170 (12.7) | 19 (9.2) |
| Intra-abdominal infection | 177 (13.2) | 33 (16) |
| Skin and soft-tissue infections | 96 (7.2) | 14 (6.8) |
| Catheter-related infection | 38 (2.8) | 14 (6.8) |
| Central nervous system | 10 (0.7) | 2 (1.0) |
| Endocarditis | 7 (0.5) | 4 (1.9) |
| Other | 85 (6.3) | 9 (4.4) |
| Considered after review not to be infected | 31 (2.3) | 3 (1.5) |
