# Supplementary material for: Microcirculatory perfusion disturbances in septic shock: results from the ProCESS trial
Source: Crit Care. 2018 Nov 20;22:308. doi: 10.1186/s13054-018-2240-5 (PMC6245723; doi:10.1186/s13054-018-2240-5)
Supplement: Supplementary file 2 — Table S2. Microcirculatory perfusion parameters by study arm. This table shows the distribution of patients and mortality rates by study arm included in the ancillary study. Overall, the mortality rates among the groups were similar. (DOCX 13 kb) [file 13054_2018_2240_MOESM2_ESM.docx]

**Additional file 2: Table S2:** MicroScan Measurement by Study Arm.

| Arm | Measurements | Cases | Death |
| --- | --- | --- | --- |
| EGDT | 135 | 63 | 13 (20.6%) |
| Protocolized Non-invasive | 141 | 65 | 10 (15.4%) |
| Control | 163 | 79 | 17 (21.5%) |
| Total | 439 | 207 | 40 (19.3%) |
