# Supplementary material for: Microcirculatory perfusion disturbances in septic shock: results from the ProCESS trial
Source: Crit Care. 2018 Nov 20;22:308. doi: 10.1186/s13054-018-2240-5 (PMC6245723; doi:10.1186/s13054-018-2240-5)
Supplement: Supplementary file 3 — Table S3. Microcirculatory perfusion analysis by study arm. In a comparison of study parameters, only MFI was found to have a statistically significant difference between study arms; however, the clinical significance of this small difference is likely minimal. (DOCX 16 kb) [file 13054_2018_2240_MOESM3_ESM.docx]

**Additional file 3: Table S3:** Microcirculation Parameters by Study Arm and Timepoint

| Biomarker | Time point | Protocol Based  EGDT Arm  Median [IQR] | Protocol Based Standard Therapy Arm  Median [IQR] | Usual Care  Arm  Median [IQR] |
| --- | --- | --- | --- | --- |
| MFI | 6 hrs  24 hrs  72 hrs | **2.73 [2.48-3.00] 2.78 [2.57-3.00]** 2.77 [2.42-3.00] | **2.87 [2.53-3.00] 2.75 [2.25-3.00]** 2.93 [2.55-3.00] | **3.00 [2.75-3.00] 2.93 [2.73-3.00]** 2.88 [2.60-3.00] |
| Total Vascular Density | 6 hrs  24 hrs  72 hrs | 21.66 [18.24-25.38] 22.57 [18.10-26.37] 21.23 [18.41-22.95] | 23.50 [20.32-26.02] 21.91 [20.00-27.19] 23.51 [21.10-25.84] | 22.63 [19.81-25.58] 23.42 [19.26-26.25] 21.78 [19.58-24.82] |
| Perfused Vascular Density | 6 hrs  24 hrs  72 hrs | 20.96 [17.22-23.68] 19.79 [17.88-24.59] 19.14 [17.16-22.22] | 20.46 [18.34-24.58] 21.03 [19.02-25.54] 22.04 [19.12-24.40] | 21.73 [18.40-24.47] 22.94 [19.16-24.59] 21.01 [18.61-24.26] |
| DeBacker Score | 6 hrs  24 hrs  72 hrs | 14.74 [12.53-16.44] 14.29 [12.39-16.69] 14.51 [12.31-16.11 | 14.68 [13.27-16.57] 14.49 [13.15-17.68] 15.61 [13.08-16.55] | 14.97 [12.94-16.57] 15.06 [13.41-17.13] 14.62 [13.0-16.21] |
| Proportion Perfused Vessels | 6 hrs  24 hrs  72 hrs | 0.90 [0.82-0.95] 0.91 [0.80-0.95] 0.91 [0.82-0.94] | 0.90 [0.84-0.95] 0.88 [0.79-0.96] 0.90 [0.85-0.96] | 0.91 [0.84-0.95] 0.90 [0.87-0.96] 0.92 [0.83-0.95] |
| Heterogeneity Index | 6 hrs  24 hrs  72 hrs | 0.30 [0.00-0.54] 0.24 [0.00-0.45] 0.22 [0.00-0.57] | 0.10 [0.00-0.39] 0.27 [0.00-0.52] 0.12 [0.00-0.45] | 0.00 [0.00-0.27] 0.00 [0.00-0.36] 0.18 [0.00-0.50] |

Supplemental Table 3: Shows the median microcirculatory perfusion parameters by arm. MFI at 6 and 24 hours was statistically significantly different between arms, driven by the EGDT versus usual care comparison. However, the clinical significance of this degree of difference is questionable. **Statistical significance p<0.05 is designated in bold.**
